# Comparative genomic analysis of enterotoxigenic *Escherichia coli* O159 strains isolated from diarrheal patients in Korea

**DOI:** 10.1186/s13099-019-0289-6

**Published:** 2019-02-21

**Authors:** Si-yun Chung, Taesoo Kwon, Young-Seok Bak, Joung Je Park, Cheorl-Ho Kim, Seung-Hak Cho, Won Kim

**Affiliations:** 1 School of Biological Sciences, Seoul National University, 1 Gwanak-ro, Gwanak-gu, Seoul, 151-742 Republic of Korea; 2 Cloud9, 133, Yeonje-gil, Osong-eup, Heungdeok-gu, Cheongju-si, Chungcheongbuk-do 28164 Republic of Korea; 3 Department of Emergency Medical Services, Sun Moon University, Asan-si, Chungcheongnam-do 31460 Republic of Korea; 4 Department of Emergency Medical Service, Pohang University, Heunghae-eup, Sindeok-ro, Pohang-si, Gyeongsangbuk-do Republic of Korea; 5 Glycobiology Unit, Department of Biological Science, Sungkyunkwan University and Samsung Advanced Institute for Health Sciences and Technology (SAIHST), 2066 Seobu-ro, Suwon, 16419 Republic of Korea; 6 Division of Bacterial Disease Research, Center for Infectious Disease Research, Korea National Institute of Health, Cheongju, 363-951 Republic of Korea

**Keywords:** Enterotoxigenic *Escherichia coli* O159, Whole genome sequencing, Virulence factors, Colonization factors, Phylo-groups

## Abstract

**Background:**

Enterotoxigenic *Escherichia coli* (ETEC) is a common cause of bacterial infection that leads to diarrhea. Although some studies have proposed a potential association between the toxic profile and genetic background, association between toxin of ETEC and phylo-group has not been reported yet. The objective of this study was to examine genomic and phylogenetic characteristics of ETEC strain NCCP15731 and NCCP15733 by whole genome sequencing and comparative genomic analysis of two phylo-groups of O159 reference strains.

**Results:**

Whole genome sequencing showed that genome size of NCCP15731 strain was 4,663,459 bp, containing 4435 CDS and 19 RNAs. The genome size of NCCP15733 was 4,645,336 bp, containing 4369 CDS and 23 RNAs. Both NCCP15731 and NCCP15733 were classified in the phylo-group A, which is one of major *E. coli* phylogenetic groups. Their serotype was O159:H34. They possessed the virulence factor such as adherence systems, auto transporter systems, and flagella segments of major driving force for ETEC pathogenicity. They also harbored STh enterotoxin. Hierarchical clustering result based on the presence or absence of a total of 108 major virulence factors of 14 O159 ETEC strains showed that seven strains in phylo-group A and seven strains in phylo-group B1 were clustered each other, respectively. Colonization factors (CFs) of NCCP15731 or NCCP15733 were not detected.

**Conclusions:**

Serotype of NCCP15731 and NCCP15733, representing major types of ETEC in Korea, was O159:H34 and their MLST type was ST218. Comparison with other O159 strains revealed that NCCP15731 was specialized for transporter system and secretion system whereas NCCP15733 had unique genes related to capsular polysaccharide. Compared with E159, the most recent common ancestor, these two strains had different toxin type and virulence factors. These results will improve our understanding of ETEC O159 strains to prevent ETEC disease.

**Electronic supplementary material:**

The online version of this article (10.1186/s13099-019-0289-6) contains supplementary material, which is available to authorized users.

## Background

*Escherichia coli* is a Gram-negative bacterium belonging to family *Enterobacteriaceae*. Most of *E. coli* are typical members of the normal microflora of the humans and animals [1]. However, they could be classified as pathogenic strains that cause serious disease like diarrhea. Pathogenic *E. coli* can be divided to intestinal pathogenic *E. coli* (IPEC) and extraintestinal pathogenic *E. coli* (ExPEC) [2]. Intestinal pathogenic *E. coli* include enteroaggregative *E. coli* (EAEC), enterotoxigenic *E. coli* (ETEC), enteropathogenic *E. coli* (EPEC) and Shiga toxin-producing *E. coli* (STEC), while extraintestinal pathogenic *E. coli* include uropathogenic *E. coli* (UPEC) depending on the various virulence genes and phenotypes that play important roles in the pathogenesis [3–8]. ETEC is a common cause of bacterial infection that leads to diarrhea in infants, young children, and travelers in developing countries [9, 10]. Because ETEC outbreaks are gained by consuming contaminated food or water, food contamination is an important concern for public health [11, 12]. ETEC can be defined by the ability to produce a heat-labile toxin (LT) and/or heat-stable toxin (ST, including STh and STp) that can disturb the intestinal secretory state, thereby causing watery diarrhea of infected patients [13–15]. While STh is produced by ETEC isolated from humans, STp originally found in pig ETEC is also associated with disease in humans [13, 16]. ETEC harbors one or more cell adhesion factors called colonization factors (CFs) to colonize epithelial cells of intestinal surfaces of hosts. ETEC CFs are named as coli surface antigens (CS) with a number except CFA/I. Currently, more than 30 colonization factors (CFs) have been identified in human ETEC. They are co-expressed with one, two, or three CFs and/or toxic factors, such as CS1 + CS3 (± CS21) with LT + STh, CS2 + CS3 (± CS21) with LT + STh, CS5 + CS6 with LT + STh, CS6 with STp, CFA/I (± CS21) with STh and CS7 with LT [17–19]. However, between 30 and 50% of ETEC have undetectable CFs, suggesting that there are still unknown CFs [20, 21]. Phylogenetic analysis is important for investigating the evolution and diversity of *E. coli* and evaluating bacterial toxicity [22]. Most commensal and pathogenic *E. coli* strains belong to phylo-groups A and B1 [23, 24]. About 90% of foodborne *E. coli* isolates in Korea belong to phylo-groups A and B1 [25].

In a previous study [26], 258 isolates from patients with diarrhea in Korea were analyzed for CFs and subjected to multi-locus sequence typing (MLST). ST171 (24%) was identified as the most prevalent ETEC type in Korea, followed by ST955, ST964, and ST656. NCCP15740 [27], representing the major MLST type ST171 of ETEC in Korea, has been investigated about its genomic features, CF genes, and virulence factors. However, other MLST types have not been investigated. In this study, we selected one of ST964 strains and one of ST656 strains identified in the previous work namely NCCP15731 and NCCP15733 [26], because these strains are the most representative strains of ST964 and ST656, and performed whole-genome sequencing. We compared whole-genome sequences of these two strains with NCCP15732 of O6 isolate and those of other ETEC strains reported as serotype O159 isolates.

## Methods

### Bacteria and strain isolation

*Escherichia Coli* were isolated from patients with diarrhea outbreak and identified as third and fourth highest prevalent MLST type (ST964 and ST656) of ETEC in Korea based on 7 isolates obtained from 2003 to 2011, respectively [26]. Candidate colonies of *E. coli* were identified based on phenotypes and biochemical properties using the API20E system (Biomerieux, Marcy l’Etoile, France). These isolated strains were deposited at National Culture Collection for Pathogens (NCCP) under the registration numbers NCCP15731 and NCCP15733. We selected 12 *E. coli* O159 strains as reference strains with the same O serotype as NCCP15731 and NCCP15733 listed in Table 2. These 12 *E. coli* O159 strains had been isolated globally from 1980 to 2011 [18]. Illumina short reads of reference strains were obtained at NCBI SRA (Sequence Read Archive) under the accession numbers listed in Table 2. De novo assemblies were performed with Spades (version 3.5.0) [28]. *E. coli* NCCP15732 was used as the reference strain because this strain represents the second highest prevalent ST955 in Korea. In comparison with 12 strains of *E. coli* O159 and NCCP15732 in Korea, we anticipated that we could identify genomic and pathogenic characteristics of NCCP15731 and NCCP15733.

### Whole genome sequencing, assembly and annotation

Genomic DNAs of a single bacterial isolate of NCCP15731 or NCCP15733 were extracted from a pure culture. Potential contamination of other microorganisms was checked using a BLAST search against non-redundant database. A sequencing library was created using TruSeq sample preparation kit (Illumina, San Diego, CA, USA). Whole genome sequencing of NCCP15731 and NCCP15733 was performed using the Illumina HiSeq 2000 platform (Theragen Etex Bio Institute, Suwon, Republic of Korea). High-quality reads were assembled by discarding low-reads, quality scores < Q20, and duplicated reads using SOAP de novo (version 1.05) [29]. Assembled contigs of NCCP15731 and NCCP15733 were annotated-using the Rapid Annotation using Subsystem Technology (RAST version 4.0) [30] server pipeline.

### Genomic analysis

The same method as described by Clermont et al. [22] was performed in silico to identify *E. coli* phylogenetic groups (A, B1, B2, D, E, and F) using Primersearch program from the European Molecular Biology Open Software Suite(EMBOSS) [31]. In silico SerotypeFinder (version 1.1) [32] was used to identify serotype of 14 O159 strains including NCCP15731 and NCCP15733 and NCCP15732. Colonization factors and MLST type were identified using CF primers [33] and *E. coli* MLST database [34]. To identify the genes encoding virulence factors, the total CDSs of NCCP15731, NCCP15732, NCCP15733 and 12 O159 strains were analyzed using BLASTP [35] against the virulence factor genes of *E. coli* listed in VFDB with an e-value of 1e−5 [36]. We selected genes with coverage of at least 60%. Resistance genes in Whole-genome sequence of all isolates were identified by ResFinder [37] Default thresholds of coverage of at least 60% and identity of at least 90% were employed.

### Phylogenetic analysis

To identify evolutionary relationship of 14 O159 strains and NCCP15732, phylogenetic analysis was performed. Multiple sequence alignments obtained from concatenated whole genome sequences of each of all strains were performed with Mugsy (version 1.2.3) [38]. The generalized time reversible + CAT model [39] was used for maximum-likelihood phylogenetic tree construction using FastTree (version 2.1.7) [40]. Resulting trees were visualized with FigTree (ver 1.3.1) (http://tree.bio.ed.ac.uk/software/figtree). In order to exclude the effect of HGT (Horizontal gene transfer) in phylogenetic analysis, multi-locus sequence analysis method was used [41, 42]. Seven housekeeping genes (*adk, fumC, gyrB, icd, mdh, purA* and *recA*) from each of all ETEC sequences were obtained according to the protocol described in *E. coli* MLST database (http://mlst.warwick.ac.uk/mlst/dbs/Ecoli/documents/primersColi_html). A phylogenetic tree based on MLST genes was created using the method employed for whole genome phylogenetic analysis.

## Results and discussion

### Genomic analysis

**Fig. 1:**
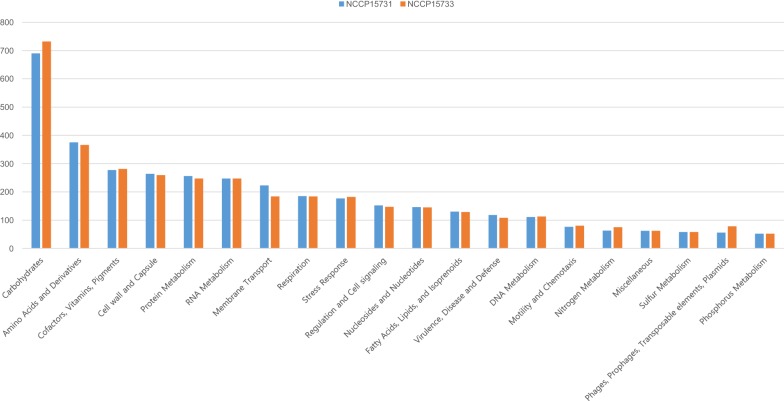
Subsystem category distribution of NCCP15731 and NCCP15733 based on the SEED databases

The genome size of NCCP15731 was 4,663,459 bp with G+C content of 50.7%. The genome size of NCCP15733 was 4,645,336 bp with G+C content of 50.6%. Based on RAST analysis, 4435 coding sequences and 19 tRNA genes were detected in the genome of NCCP15731, of which 3855 (87%) were functional. A total number of 4369 coding sequences and 23 tRNA genes were detected in the genome of NCCP15733, of which 3852 (88%) were functional (Fig. 1). In silico analysis revealed that serotype of both NCCP15731 and NCCP15733 was O159:H34 belonging to phylo-group A, one of major *E. coli* phylogenetic groups [23]. Genomic and phylogenetic characteristics of NCCP15731 and NCCP15733 were shown in Table 1. We investigated toxin types and colonization factors of NCCP15731 and NCCP15733. Both NCCP15731 and NCCP15733 possessed STh enterotoxin, but not LT enterotoxin. As shown in Table 2, two strains, E133 and E1679sc, contained both STp and LT. Four strains contained STh or STp, while seven strains contained LT. CFs were not found in eight strains of all isolates, including NCCP15731 and NCCP15733. NCCP15732 had STh and LT enterotoxin and harbored CS1 and CS3 of CFs.

**Table 1:** Genomic and phylogenetic characteristics of NCCP15731 and NCCP15733

Strain	NCCP15731	NCCP15733
Genome size	4,663,459	4,645,336
% GC	50.7	50.6
N50	9457	14,126
L50	147	105
Total contigs	986	694
Total subsystems	587	588
Total CDS	4435	4369
tRNAs	19	23
Serotype	O159:H34	O159:H34
Phylo-group	A	A

**Table 2:** Genomic features of whole genome datasets of Enterotoxin *Escherichia coli* strains used in this study

Strain	Genome (Mb)	Phylo-group	MLST type	Serotype	Toxin type	CF type^1−2^	Accession number
*E. coli* NCCP15731	4.66	A	218	O159:H34	STh	CF NEG	QICG00000000
*E. coli* NCCP15733	4.64	A	218	O159:H34	STh	CF NEG	QICF00000000
*E. coli* E159	4.95	A	218	O159:H34	LT	CF NEG	ERS077713
*E. coli* E133	4.89	A	10	O159:H4	LT + STp	CS12	ERS044470
*E. coli* E1532	4.83	A	10	O159:H4	LT	CF NEG	ERS077694
*E. coli* E1573	4.95	A	10	O159:H4	LT	CF NEG	ERS077704
*E. coli* E1679sc	4.95	A	10	O159:H4	LT + STp	CS12	ERS163315
*E. coli* E391	4.91	B1	641	O159:H21	LT	CF NEG	ERS077592
*E. coli* E833	5.09	B1	641	O159:H34	LT	CS28a	ERS077628
*E. coli* E885	4.96	B1	641	O159:H21	LT	CF NEG	ERS077638
*E. coli* E1640	5.01	B1	641	O159:H34	LT	CF NEG	ERS077734
*E. coli* E5085	5.06	B1	1490	O159:H34	STp	CS6	ERS055670
*E. coli* E5088	5.07	B1	1490	O159:H34	STp	CS6	ERS055673
*E. coli* E5051	5.25	B1	295	O159:H18	LT	CS6	ERS055664
*E. coli* NCCP15732	4.80	A	4	O6:H16	LT + STh	CS1 + CS3	–

All strains except NCCP15731 and NCCP15733 were referred to this paper [17]

### Phylogenetic analysis

**Fig. 2:**
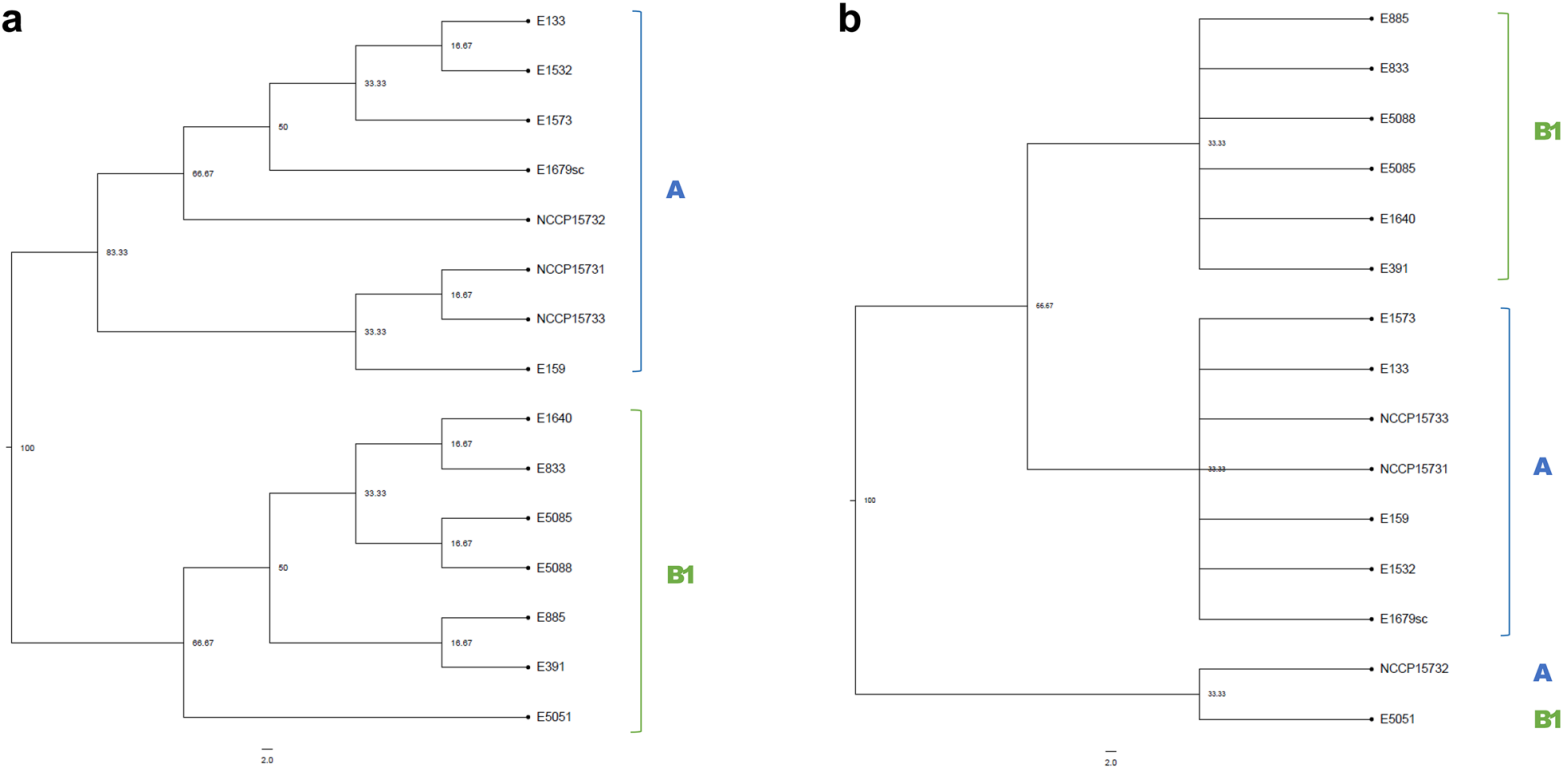
Phylogenetic tree of NCCP15731 and NCCP15733. **a** Whole genome phylogenetic tree, **b** MLST phylogenetic tree. The trees were obtained by approximately-maximum-likelihood analysis with a GTR (generalized time-reversible) + CAT model of concatenated alignments of whole genome sequences and MLST genes. Evolutionary time is adjusted to 100. A lower value means that it has been relatively recently branched. The scale bar indicates 2.0 substitutions per site

Phylogenetic comparison of candidate genes implemented in SEED [43] showed that NCCP15731 and NCCP15733 were most close to *E. coli* O157:H7 str. 88.1467 (score: 531 and 530, respectively). Whole genome phylogenetic analysis and MLST phylogenetic analysis were performed based on multiple sequence alignments of whole genomes, and seven MLST genes (*adk*, *fumC, gyrB, icd, mdh, purA,* and *recA*) of 15 *E. coli* isolates including NCCP15731, NCCP15732 and NCCP15733, respectively (Fig. 2). Whole genome phylogenetic tree showed that NCCP15732 and 14 *E. coli* O159 strains were clustered into two phylo-groups (A and B1) like previous study [18]. NCCP15731 and NCCP15733 in this study belonged to phylo-group A and were clustered with *E. coli* O159:H34 str. E159 with sequence type of ST218 (Fig. 2a). MLST phylogenetic analysis also showed that NCCP15731 and NCCP15733 belonged to phylo-group A (Fig. 2b). *E. coli* E159 strain was also placed with NCCP15731 and NCCP15733 in MLST phylogenetic tree.

### Virulence factors

The acquisition of virulence factors has been suggested to be a major driving force for ETEC pathogenicity [44, 45]. ETEC causes disease by colonizing the small bowel through attachment to the host epithelial lining by surface proteins called CFs and possibly other surface structures. Subsequently, adherent ETEC elaborates enterotoxins that cause typical clinical manifestations of ETEC-induced diarrhea. Thus, virulence factors can be used as important guide for understanding their pathogenicity. Virulence factors of NCCP15731 and NCCP15733 were investigated and these factors were compared with those of 14 O159 strains and NCCP15732. We identified 222 virulence factors grouped into 28 categories and 74 subcategories (Additional file 1: Table S1).

**Fig. 3:**
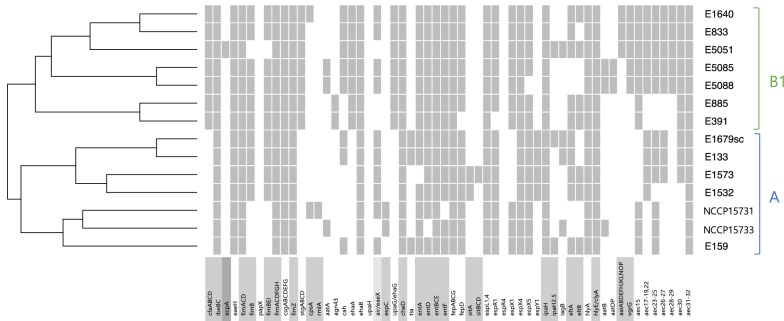
Hierarchical clustering of 14 ETEC strains according to virulence factors. The dendrogram and associated heatmap based on the presence or absence of 108 major virulence genes were constructed using R version 3.4.3 [43]. Grey and white colors indicate gene presence and gene absence, respectively

Among O159 strains, a total of 125 virulence genes were found in all of 14 ETEC strains. Each of NCCP15731 and NCCP15733 had 150 (67.6%) of these 222 virulence genes, respectively. They had the least number of virulence factors among 14 *E. coli* strains used in this study. In silico analysis revealed that major virulence factors belonged to the following six categories: adherence, auto transporter, iron uptake, non-LEE encoded TTSS effectors, toxin, and secretion system. Hierarchical clustering based on the presence or absence of 108 major virulence genes of 14 O159 strains was constructed using R (version 3.4.3) (Fig. 3) [46]. 14 O159 strains of phylo-group A and B1 shown in Fig. 2a again completely clustered each other, respectively. These results suggested that the virulence factors were related with the phylo-group [23, 44, 47–50].

ECP (*E. coli* Common Pilus), *EaeH*, Type I fimbriae, Curli fimbriae, Stg fimbriae, AIDA-I type and *UpaG*, which belong to 13 potentially functional systems in adherence and auto transporter categories, are known to be produced by pathogenic *E. coli* [51]. These functional systems suggest that the ability of ETEC to attach to the host surface is the most important step in successful colonization [52]. Compared with NCCP15732 strain, all of O159 strains had *EaeH* system in adherence category. A virulence factor, *espC*, was only identified in NCCP5731 and NCCP15733. Conversely, *ehaA* auto transporter gene and stg fimbriae (*stgABCD*) systems were only found in phylo-group B1. It is known that stg fimbriae (*stgABCD*) systems contribute to the attachment of human epithelial cells. They are associated with phylogenetic group B1 [53].

Regarding toxins, enterobactin synthesis (*entABCE*) and ferric-enterobactin tansport (*fepABCG*) were present in all of the O159 strains. Alpha-hemolysin related gene (*hlyA)* plays a major pathogen role in ETEC and other pathogenic *E. coli* strains [54]. It was present in all O159 strains. Heat-labile enterotoxin(LT), i.e., *eltA* (10/14, 71.4%) and *eltB* (7/14, 50.0%) were found in O159 strains [55]. However, none of these heat-labile genes were found in NCCP15731 or NCCP15733, while NCCP15732, another strain in Korea, had two heat-labile genes.

### Resistance factors

All isolates in the study were analyzed for antimicrobial susceptibility. We identified 11 resistance factors of six phenotypes (Table 3). One resistance factor, *mdf(A)* of MLS (Macrolide Lincosamide and Streptogramin) phenotype was found in all of isolates. We identified 11 resistance factors of six phenotypes (Table 3). NCCP15731 had six resistance factors of six phenotypes, but NCCP15733 had only one resistance factor. In phylo-group A, except NCCP15731 and E1573, five strains had only a *mdf(A)* resistance factor of MLS phenotype, while seven strains in phylo-group B had at least three to a maximum of seven resistance factors. NCCP15732 of O6 serotype had only a *mdf(A)* resistance factor of MLS phenotype and any of resistance factors of this strain were not found.

**Table 3:** Genotypic and phenotypic antimicrobial resistance factors of Enterotoxin *Escherichia coli* strains used in this study

Strain	Aminoglycoside			Beta-lactam	MLS		Sulphonamide		Tetracycline	Trimethoprim	
	*aadA1*	*aph(6)*-*Id*	*aph(3′’)*-*Ib*	*blaTEM*-*1B*	*mdf(A)*	*mph(A)*	*sul1*	*sul2*	*tet(A)*	*dfrA1*	*dfrA8*
NCCP15731	+	−	−	+	+	−	+	−	+	+	−
NCCP15733	−	−	−	−	+	−	−	−	−	−	−
E159	−	−	−	−	+	−	−	−	−	−	−
E1532	−	−	−	−	+	−	−	−	−	−	−
E1679sc	−	−	−	−	+	−	−	−	−	−	−
E133	−	−	−	−	+	−	−	−	−	−	−
E1573	−	+	+	+	+	−	−	+	−	−	+
NCCP15732	−	−	−	−	+	−	−	−	−	−	−
E5051	+	−	−	+	+	−	+	+	+	+	−
E5085	−	−	−	+	+	+	−	−	−	−	−
E5088	−	−	−	+	+	+	−	−	−	−	−
E391	−	+	+	+	+	−	−	+	−	−	+
E833	−	+	+	+	+	−	−	+	+	−	+
E1640	−	−	−	−	+	−	−	−	−	−	−
E885	−	+	+	+	+	−	−	+	+	−	+

### Comparison with other *E. coli* strains in phylo-group A

To calculate Average Nucleotide identity (ANI), five genomes of O159 of phylo-group A including NCCP15731 and NCCP15733 were compared with each other using ANIu calculator [56]. *E. coli* E1573, and E1679sc were excluded due to the presence of strains with the same MLST type and toxin type. Results indicated that NCCP15731 was most similar to NCCP15733, with OrthoANIu value of 99.97% (Table 4). Both NCCP15731 and NCCP15733 were most similar to *E. coli* strain E159 among reference strains in phylo-group A, with OrthoANIu values of 99.90% and 99.91%, respectively.

**Table 4:** Average nucleotide identity values based on USEARCH

	NCCP15731	NCCP15733	E159	E133	E1532
NCCP15731	–	99.97 (73.04)	99.90 (72.79)	99.15 (68.37)	99.23 (67.29)
NCCP15733	99.97 (71.21)	–	99.91 (73.34)	99.18 (70.64)	99.15 (68.57)
E159	99.90 (62.93)	99.91 (65.03)	–	98.99 (72.80)	99.00 (70.40)
E133	99.15 (59.53)	99.18 (63.08)	98.99 (73.32)	–	99.87 (73.95)
E1532	99.23 (59.38)	99.15 (62.06)	99.00 (71.87)	99.87 (74.95)	–

Numbers indicate OrthoANIu value (%) and numbers in parentheses indicate the genome coverage (%)

**Fig. 4:**
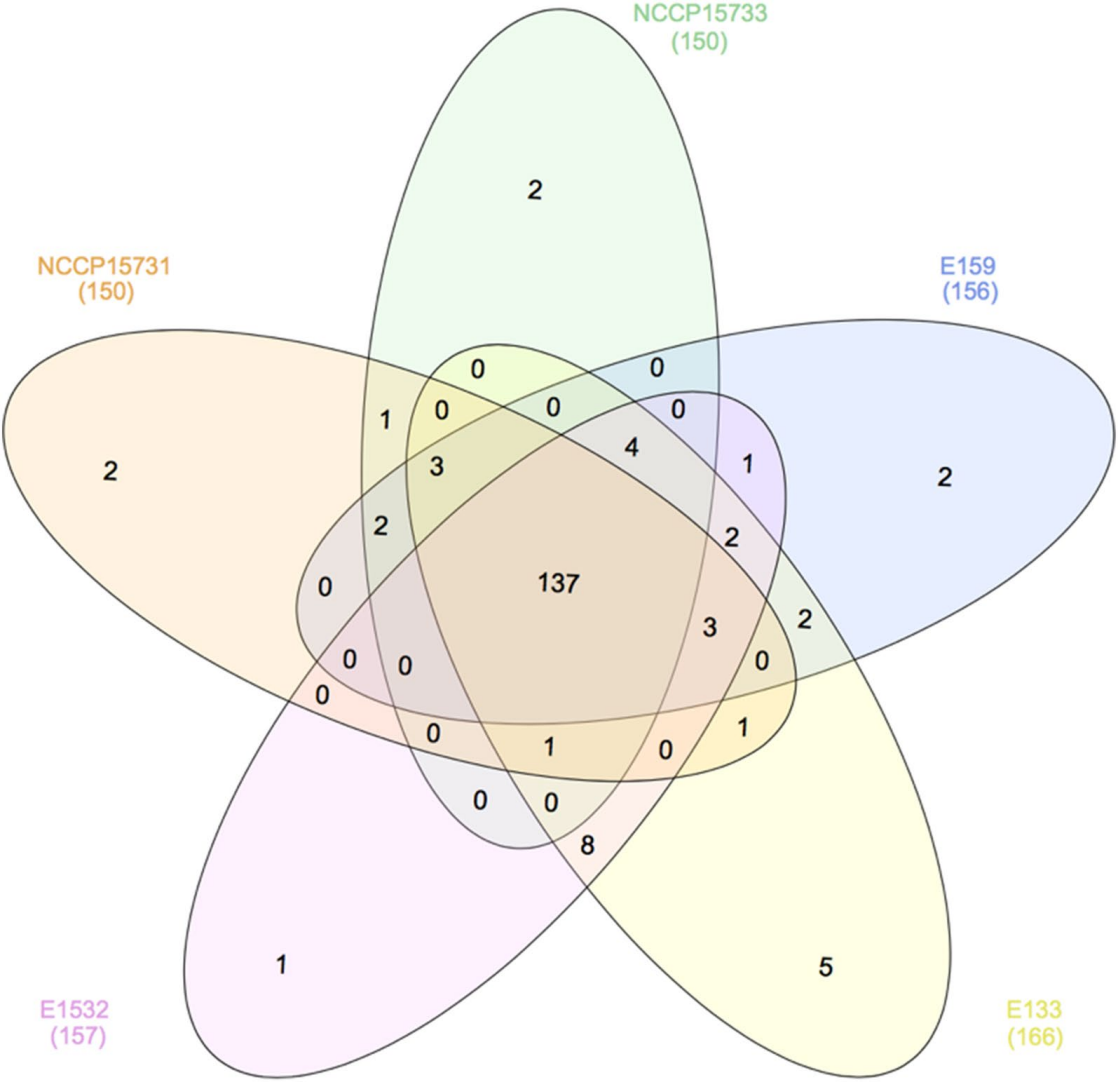
Comparison of virulence factors of five ETEC strains in phylo-group A

Venn diagram of the virulence factors of the five strains including NCCP15731 and NCCP15733 in phylo-group A was obtained using InteractiVenn [57]. They shared 137 genes encoding *E. coli* common pilus (ECP) proteins, flagella-biosynthetic proteins, enterobactin transport proteins, and proteins related to Type II secretion system (Fig. 4). Comparison of NCCP15731, NCCP15733, and E159 (the most recent common ancestor of NCCP15731 and NCCP15733) showed that these three strains shared 142 genes. Plus, NCCP15731 and NCCP15733 shared *espC* gene related to auto transporter and *orgA* gene encoding bsa T3SS secretion system. Compared to NCCP15733 and E159, NCCP15731 had three unique virulence genes: *aatA* encoding auto transporter protein, *aatB* encoding ABC transporter protein, and *iagB* encoding TTSS related to secretion system. Compared to NCCP15731 and E159, NCCP15733 had two unique virulence genes: *cpsA* and *rmlA* encoding capsular polysaccharide. Moreover, these two unique genes were not found in other *E. coli* O159 reference strains. In enterobactin synthesis of virulence systems, *entF* gene was found in NCCP15731, but not in NCCP15733 whereas *entD* gene was found in NCCP15733, but not in NCCP15731.

## Conclusions

*Escherichia coli* NCCP15731 and NCCP15733, previously identified as MLST types ST964 and ST656, respectively, were isolated from diarrheal patients. However, multi-locus sequence typing (MLST) profiles showed that the MLST type of each of two strains was ST218. NCCP15731 and NCCP15733 belonged to phylo-group A and their serotype was O159:H34. In both whole genome and MLST phylogenetic analyses, NCCP15731 and NCCP15733 also belonged to phylo-group A. Hierarchical clustering based on the presence or absence of major virulence factors suggest that the virulence factors are associated with the phylogenetic group. In comparison of virulence genes of 14 strains, NCCP15733 has unique genes related to capsular polysaccharide. NCCP15731 has no unique virulence gene. However, in comparison with NCCP15731 and E159, it showed differences in the auto transporter, ABC transporter, and secretion system. Genomic analysis of NCCP15731 and NCCP15733 will be useful for further study on the development of ETEC vaccines.

### Future directions

In summary, serotype and MLST type of NCCP15731 and NCCP15733 were O159:H34 and ST218, respectively. Unlike other O159 strains, CF gene of NCCP15731 and NCCP15733 was not detected. These strains have unique genes related to capsular polysaccharide, auto transporter, and secretion system. Moreover, both strains do not contain LT genes. These results will improve our understanding of ETEC O159 strains to prevent ETEC disease. However, because the results were obtained from in silico analysis, experimental confirmation of these results is required.

## Additional file

**Additional file 1.** List of virulence factors of 16 ETEC O159 strains.

## Abbreviations

ETEC: enterotoxigenic *Escherichia coli*
LT: heat-labile toxin
STh: heat-stable toxin
MLST: multi-locus sequence typing
RAST: Rapid Annotation using Subsystem Technology
CDS: coding DNA sequences
CF: colonization factor
NCCP: National Culture Collection for Pathogens
